# Does the selection of high-quality scenic spots promote the growth of tourism economy? evidence from China’s 5A-rated tourist attractions

**DOI:** 10.1371/journal.pone.0304108

**Published:** 2024-06-10

**Authors:** Qingyong Xu, Xu Cheng, Hehua Zhao

**Affiliations:** School of Tourism, Nanchang University, Nanchang, Jiangxi, China; Zhongnan University of Economics and Law, CHINA

## Abstract

To stimulate the regional tourism economy, local governments often seek to increase the number of 5A-rated tourist attractions. However, there have been few analyses examining the economic benefits and influence mechanisms of 5A-rated attraction selection. Using the quality signaling theory and data from 282 prefecture-level cities spanning 2002 to 2019, this study examines the impact of 5A-rated attraction selection on the local tourism economy with the difference-in-differences method. This study’s results demonstrate that the selection of 5A-rated attractions significantly contributes to the growth of the local tourism economy. The robustness test results confirm the validity of this conclusion. A mechanism analysis reveals that 5A-rated attractions positively impact the tourism economy via investments in infrastructure, popularization of informatization, and increased external openness. Furthermore, the study suggests that the effect of 5A-rated attractions is more pronounced in economically underdeveloped regions and low-level cities. The results of this study contribute to the sustainable development of China’s tourism economy and may provide guidance for the establishment of tourism evaluation systems in other international locations in order to foster economic growth.

## 1 Introduction

The tourism industry is recognized as one of the largest and fastest-growing sectors globally, demonstrating substantial linkage effects that contribute to national economic growth and restructuring. Prior research has confirmed the vital role tourism plays in employment generation, poverty reduction, foreign exchange augmentation, infrastructure development, and income disparity reduction [1–4]. The industry’s exemplary performance has drawn attention to tourism destinations, leading to the proliferation of rankings and evaluations. To foster advancement, stimulate the market, enhance segmentation and management of tourist attractions, improve standardization, and support long-term development [5], the Chinese government has established a rating system for tourism destinations. This system includes the selection of A-level scenic spots, an initiative actively endorsed by the government. The 5A-rated tourist attractions (5ATAs) are recognized as the zenith of quality classification, symbolizing China’s premier tourist spots [6]. It’s among the most influential, highly recognized, and strictly judged ratings, frequently cited as a quality signal in an asymmetric information tourism market. Many attractions strive for continuous enhancement to gain a 5A rating, thereby elevating their reputation and tourism revenue, fostering local economic growth [1]. However, the specific pathways through which 5ATAs selection affects tourism economy have not yet been fully substantiated, warranting further investigation.

The 5ATAs selection process is a product of China’s tourism evolution. Economic globalization has escalated global competition for tourism destinations, resulting in a diverse and abundant market yet plagued by inconsistency and mismanagement [7]. With a late start but rapid growth, China’s tourism industry now boasts over 30,000 various attractions, propelling destinations to seek competitive advantages through quality disclosure and rating [8]. The 5ATAs emerged amid high expectations, initially launched in 1999 and expanded to the 5A rating in 2007. Following the implementation of the ’Classification and Evaluation for Scenic Spots’ Quality Grades (GB/T 17775–1999)’ in 1999 [6], the Chinese government has made multiple revisions to the relevant documents. The evaluation system encompasses three key components: service and environmental quality, landscape quality, and tourist opinion scoring. This multifaceted approach necessitates enhanced hardware facilities, deeper cultural significance, and superior service quality at the scenic location. By the end of 2019, a total of 280 5A-level scenic spots were established across 31 provinces on the Chinese mainland. But issues such as commercialization and arbitrary pricing have arisen. In response, the Chinese government strengthened regulation, even revoking or warning several A-rated sites, including 5A-rated ones, from 2015, 2016, and 2019. After nearly two decades of growth, China has become the world’s largest outbound and domestic tourism country and the third-largest inbound tourism recipient. According to the World Economic Forum’s Tourism Competitiveness Report of 2022, China ranks 12th globally in tourism competitiveness. In 2019, pre-pandemic, China recorded 6.63 trillion yuan in total tourism revenue, contributing 10.94 trillion yuan to GDP, accounting for 11.05%. Amidst this rapid economic growth, the role of rating activities has come under increased scholarly focus due to its integral role in the industry’s progression.

The practice of evaluating tourism activities manifests the application of quality signaling theory within the tourism industry. Quality signals can convey brand information in the product market with asymmetric information, which is particularly important for the market that focuses on service [9]. Quality signals induce differentiation effects, where honorary labels notably enhance the perception of service quality, visitor satisfaction, and destination reputation [10]. They allow for the delineation of tourism destinations across varying levels and quality [11]. Authoritative third-party validation and rating confer increased attention and recognition to tourism sites [12], strengthening the market competitiveness of high-quality attractions and extending their market impact. With the advancement of network technology, quality signals exert a more profound influence on travel choices, enabling tourists to filter for superior destinations through labels [10]. Although certification and evaluation can confer prestige and benefits to sites, the economic impacts and underlying mechanisms of 5ATAs evaluations on tourism still necessitate further investigation.

In light of this, the present study, grounded in quality signal theory, employs a difference-in-differences (DID) approach to analyze data from 282 prefecture-level cities spanning 2002 to 2019, probing the influence of 5ATAs selection on local tourism economy. The findings reveal that the 5ATAs selection process exerts a significant promotional effect on the local tourism economy. This conclusion remains robust after parallel trend testing, excluding sub-provincial cities, substituting explanatory variables, shortening the time sample, conducting placebo testing, and treating tail values of the dependent variable. Mechanism tests indicate that 5ATAs selection positively impacts local tourism economics through enhanced infrastructure investment, informatization popularization, and external openness. Additionally, we discerned that the promotive effect of 5ATAs selection is more pronounced in economically underdeveloped regions and lower-tier cities.

The principal contributions of this study can be delineated into four aspects. First, from a research perspective, this study employs the 5ATAs selection as a policy shock, using data from 282 prefecture-level cities between 2002 and 2019 to investigate the effect of 5ATAs selection on tourism economics. It concludes that the 5A rating can stimulate local tourism economic growth, thereby enriching the body of research in related fields. Second, on the theoretical plane, this article integrates quality signal theory into tourism, providing a theoretical foundation for the investigation of tourism evaluation activities, and analyzing the benefits of site selection policy, thereby extending the application of quality signal theory within the tourism domain. Third, concerning research content, the study explores not only the effect of 5ATAs selection on local tourism economy but also examines possible underlying mechanisms, discovering that such selection, through enhancing infrastructure investment, informatization popularization, and external openness, advances local tourism economic growth. This provides a comprehensive assessment of the intrinsic mechanisms and transmission paths of 5ATAs selection’s influence on tourism economic development. Fourth, regarding research significance, the study, through heterogeneity testing, uncovers that the stimulating effect of this selection policy is more pronounced in economically underdeveloped areas and lower-tier cities. These insights may guide the rational inclination of policy and facilitate the rapid development of the tourism economy.

The paper is structured into six main sections. Following this introduction, the second section reviews relevant literature and posits hypotheses. The third section delineates the research methodology, variable definitions, and data utilization. The fourth section is dedicated to result analysis, comprising baseline regression followed by robustness testing, mechanism examination, and heterogeneity testing. The fifth section discusses the empirical results. The sixth section summarizes the study, deriving insights, and highlighting limitations and future prospects.

## 2 Literature review

### 2.1 Quality signal theory

Quality signal refers to the phenomenon where, in a product market characterized by information asymmetry, a product or brand exhibits distinct signal characteristics. Such products often employ specific methods to convey quality signals, thereby achieving an enhanced brand effect and augmenting their market competitiveness through increased product pricing [13]. Quality signals facilitate product differentiation, enabling the distinction of products of varying quality via signs, labels, and grades. Utilizing third-party ratings, high-quality products can be more distinctly marked, thereby mitigating market information asymmetries [9,11,12]. In the context of China’s economic growth and the burgeoning tourism industry, a surge in destination offerings has occurred. This expansion and diversity, along with heightened competitiveness, have intensified the competition, especially among similar scenic spots. In this intricate tourism market, quality signals become crucial for tourists in making informed travel decisions.

Quality information is commonly deduced through external observation [10]. Tourism destinations possess various quality attributes, such as infrastructure, cultural content, and service quality. Due to the disconnect between tourists and destination markets, objective and accurate understanding is difficult to attain prior to visitation [14], complicating efficient and high-quality choices [15]. Previous studies indicate that if the transmission of quality signals is hindered, the resultant benefits are minimal. However, when multiple forms of quality signals are recognized by potential customers, the benefits significantly increase [9,12]. To effectively convey clear and authoritative quality signals to tourists, the involvement of an independent third party for quality disclosure is essential [10]. Third-party quality assessments are generally perceived as more reliable and credible by tourists compared to those issued by the tourist destinations themselves. This enhances the transparency of the tourism market by providing information that assists consumers in distinguishing between different destinations, thereby facilitating a better match between tourists and destinations [11,12]. Quality signals, conveyed through labels, honors, and grades, stratify products and serve as crucial references in destination selection [9]. In this study, the external evaluator is identified as the government, which possesses significant authority, earns the trust of tourists, and has the capability to disseminate quality signals broadly, thereby aiding in the branding of tourist attractions. The 5A grade certification for tourist spots conveys a potent quality signal to the public [11,16], indicating an official endorsement of the site [11,13]. Quality signaling theory is well-suited to the 5ATAs selection policy, providing substantial theoretical backing for this research. While this theory has seen widespread use in sectors like food and finance, its application in tourism has been limited. This study aims to bridge this gap by offering insightful contributions to the field of tourism research.

### 2.2 Tourism economy

Since the initiation of the Reform and Opening-up policy, the Chinese government has shifted its focus toward economic development. Official promotions have become closely tied to economic performance, which has comprised a significant portion of assessments, spurring local governments to maximize economic growth efforts [17,18]. Beginning in the early 1990s, China has annually attracted a substantial number of international tourists [19], and the consequent growth in foreign exchange earnings and employment opportunities has progressively increased attention on the development of the tourism industry. Local governments have exhibited robust enthusiasm toward this end, and both the tourism industry and tourism economics have experienced rapid development during this period [20]. With the elevation of the tourism industry’s status and an increase in its proportion within the overall economy, the development of tourism and tourism economics has attracted greater research interest [21]. In many regions, the tourism sector has displayed a remarkable contribution to economic growth [20,22–25], and the rapid advancement of tourism economics has shifted policy evaluation and tourism economic development level measurement into the focus of public attention.

Investigating the relationship between governmental policies and tourism economics is instrumental for evaluating policy effectiveness, fostering policy benefits, and promoting tourism economic development. It serves as both a reference and a guide. Scholars have explored the influence of the Western Development Strategy on tourism development, concluding that it has effectively stimulated the growth of tourism economics [26]. Similar studies have validated the diverse effects of tax policies on tourist arrivals in different countries [27], and the management of the ecological environment has been shown to promote the sustainable growth of the tourism industry, with debt financing playing an intermediary role [28]. Additionally, research into the impact of Swiss official development assistance on the number of visits and hotel accommodations from recipient countries found that international aid favorably boosts tourism numbers and expenditure in the aid-giving country [29]. Moreover, policies concerning climate [30], ecological preservation [31], economics[26,27,32–34], openness [35,36], public affairs [26,37], cultural-tourism integration [38,39], and high-speed rail commencement [40,41] have also been proven to influence tourism demand and economics. As the most rigorous and highest standard of selection policy in China, the study of the relationship and underlying mechanisms between the 5ATAs selection and tourism economics holds significant implications for the development of China’s tourism industry.

Tourism economics serves as a vital gauge of the development of the tourism industry, affording an immediate illustration of the benefits generated therein. Previous studies have deployed measures such as per capita domestic and international tourism revenue to represent the level of tourism economic development [10,17]. Other scholars have utilized domestic and international tourist numbers and revenues to assess the growth of tourism economics [38]. When exploring the influence of the Western Development Strategy on tourism economics, researchers have observed the development of tourism economics by examining the increase in the percentage of total tourism revenue in GDP [26]. In assessing the impact of high-speed rail on tourism economics, the article employed measures such as total domestic tourism revenue, the number of domestic tourist arrivals, and per capita domestic tourism revenue to symbolize tourism economics [41]. In the present study, the assessment of tourism economic development is conducted using per capita domestic and international tourism reception numbers and revenues. By distinguishing between domestic and international visitor markets, and focusing on both per capita reception numbers and per capita tourism revenues, this approach provides a more scientific and comprehensive measurement of arrival and consumption scenarios. This contributes to a more accurate examination of the relationship between 5ATAs selection and tourism economics.

### 2.3 Destination selection policy

Tourism destinations play a pivotal role in tourism activities, and the evaluation and ranking of such destinations constitute a key area of inquiry in tourism research [42]. With the advent of the new millennium, honorary titles and official accreditations for tourism destinations have garnered increased attention from tourists, transforming the evaluation and rating of cities and attractions into a burgeoning research hotspot. Scholars focusing on China’s best tourist cities have deduced that such ratings can enhance regional tourism economics [17], while empirical studies have unveiled the promotional effects of civilized city evaluations on tourism development, with notable short-term and long-term variances [10]. Within the domain of attraction rating and accreditation, World Heritage assessment has assumed a prominent position. Research conducted on the Chinese market concluded that World Heritage status exerts a noticeable positive impact on international tourist arrivals [19,43]. Additionally, an analysis of World Heritage selection policies in relation to visitor numbers provided categorical verification of the promotional effects of cultural and natural heritage on tourism economics in different developmental stages of countries [44]. The conclusions are not unequivocal; some scholars argue that the impact of World Heritage Site (WHS) selection, influenced by various factors, acts merely as a "placebo" effect [44], and may even induce negative consequences [45]. Instances include local residents in Shirakawa-go, Japan, experiencing diminished happiness due to World Heritage designation [46], and no improvement in tourism destination performance in Italy and Macau following World Heritage listing [45]. Currently, articles investigating the relationship between 5ATAs selection and tourism economics are scarce, and research on the economic benefits produced by this policy is not exhaustive.

The 5ATAs, esteemed as ’world-class’ top-tier scenic destinations, represent the highest official recognition in China and symbolize the nation’s prominent international stature. These attractions adhere to stringent selection criteria and uphold a robust social reputation [19,47]. According to the Management Measures, the selection of these esteemed scenic spots aims to elevate service and management standards, foster a positive image for tourist attractions, and contribute to the sustainable growth of the tourism economy. In recent times, 5ATAs have witnessed rapid development, escalating influence, expanding market share, and a notable increase in economic contributions, positioning it as an indispensable segment of China’s tourism market [6]. Consequently, analyzing the selection criteria of 5ATAs holds significant implications for advancing local tourism economy.

In the current scholarly context, research on the impact of the 5A rating policy on tourism economic growth is scarce. A closely related study is that of Yang and Lin et al. (2010), which utilized the DID approach to discuss the impact of World Heritage sites and 4A and 3A level scenic areas on the tourism economy [19]. This study diverges from the aforementioned study in four key aspects. First, while previous research predominantly focused on the influence of World Heritage designations on the influx of international tourists, giving limited attention to the impact of 4A and 3A level scenic areas on the tourism economy, this study delves more specifically into the effects of the 5A level scenic area rating policy on tourism economy. Second, the previous study used annual provincial data from the period 2000 to 2010, whereas the current study employs data from prefecture-level cities spanning from 2002 to 2019, offering more up-to-date, precise, and persuasive data. Third, this study incorporates the theory of quality signaling, amalgamating theory with empirical content, thereby enhancing the academic merit of the work. Finally, building upon the foundations of previous research, this study also delves into the underlying mechanisms.

Recent studies have revealed that 5ATAs, owing to their unique characteristics and developmental needs, contribute to the reduction of urban pollutant emissions [6,48]. Similarly, research indicates that 5A selection activities have led local governments and relevant authorities to enhance environmental oversight, effectively improving environmental conditions [47]. Another scholar used online review data based on machine learning to explore tourists’ satisfaction with 5A-level geological scenic spots [7].However, the influence of 5ATAs selection on tourism economics is still a relatively unexplored domain, warranting more comprehensive research.

### 2.4 Hypothesis presented

In the context of tourism activities, the selection of a destination is often the initial and most crucial step. The tourism destination market harbors multiple quality attributes that may not be readily perceptible, necessitating a label to expedite the identification of high-quality offerings [10]. In China, 5ATAs, certified by government authorities, represent the pinnacle of tourist attractions [6], and stand as one of the most significant labels and signals within the tourism destination market. Guided by Quality Signal Theory, the labeling and quality disclosure of tourist areas possess an allure for tourists [42], capable of enhancing both the reputation and competitiveness of an area, subsequently influencing tourist travel choices and expenditure. Such evaluation and rating policies manifest differential impacts on tourism economics across various regions [49], and their effectiveness may vary [19]. As a widely concerned selection policy, whether 5A scenic spots follow the quality signal theory and play a due driving role is worthy of in-depth exploration. Based on this, the following hypotheses are proposed:

H1: The selection of 5ATAs has promoted the development of local tourism economics.

Tourism showcases pronounced interconnectivity, maintaining close associations with other industries and domains [50]. This implies that during the process wherein 5ATAs influence tourism economics, other factors might intercede. Infrastructure construction bears significant implications for the development of tourism [1,26,48], and aspects such as transportation convenience, the completeness of auxiliary services, and the quality of travel experiences will affect tourists’ arrival and spending desires[26,51]. Infrastructure investment is likely to influence tourist experience and choices, and may play a role in the impact of 5ATAs selection policies on tourism economics. Since the onset of the new millennium, the rapid advancement of information technology and the gradual ubiquity of the internet as a primary means of communication, information acquisition, and payment have emerged [52]. Coupled with Quality Signal Theory, governments have differentiated various quality tourism areas through evaluation and rating activities, attaching labels to different quality tourist destinations [17]. Such labels require dissemination to the public, thereby forming a brand and enhancing competitiveness. The higher the level of informatization and internet penetration, the stronger the quality signal effect [10], the better the brand effect, and the higher the service quality [53], thus increasing the attraction to tourists. Furthermore, the selection criteria for 5ATAs also assess the infrastructure and information level of the area and locality, measuring basic service capabilities and tourist experience. The degree of openness is a vital criterion for attracting and utilizing foreign investment and represents an important manifestation of the globalized high-quality development of a scenic area [36]. Greater openness can attract foreign investment, garner support for scenic area construction, and amplify its reputation and influence abroad, releasing strong quality signals on a broader scale, possibly aiding in the promotion of tourism economic development in 5ATAs. Based on this, the following hypothesis is introduced:

H2: Infrastructure investment, the level of informatization, and the external openness have served as mediating factors in the impact of 5ATAs selection on tourism economics.

It is universally recognized that China encompasses a vast territory, and this leads to considerable variations in tourism resources across different regions, reflecting the disparities in economic development levels and the heterogeneity in the growth stages and sizes of individual cities [2]. Insights gleaned from the selection criteria for 5ATAs reveal that emphasis is placed on infrastructure construction, quality of services within the scenic area, and tourist satisfaction. Geographically, China’s population and attractions are characterized by an east-west dichotomy, where the eastern regions are more densely populated with broader markets, greater consumer purchasing power, and more robust local governmental support for the development of 5ATAs. These factors contribute to an enhancement in service quality, ensuring tourist satisfaction, but also engendering intensified competition among scenic areas [10]. Conversely, the central and western regions boast unique resources but face challenges in market access, funding, and service capabilities [26]. Even within the same province, variations are observed among different prefecture-level cities. Some cities benefit from strategic geographical locations, convenient transportation, quality basic services, substantial population, robust industrial backing, and strong consumer purchasing power. These attributes enable a more effective utilization of favorable policies. Conversely, some prefecture-level cities still grapple with developmental inadequacies and challenges. Economically prosperous regions and higher-tiered cities may possess an augmented ability to establish premium scenic areas, cultivate brands, and thus attract tourists [41]. This influences the economic stimulatory role of 5ATAs. Based on this understanding, the following hypothesis is posited:

H3: The stimulatory effect of 5ATAs manifests divergent characteristics across regions with varying economic development levels and cities of different tiers.

## 3 Methods and data

### 3.1 Econometric model

The DID method represents a quasi-experimental paradigm extensively employed in empirical research to assess the ramifications of governmental enactments or particular interventions. Through an analytical comparison of outcome variable fluctuations pre- and post-policy enactment, the DID methodology elucidates the quantifiable impact of policy interventions on economic outcomes [10,17]. The evaluation of 5ATAs affords a rare "quasi-natural experiment" opportunity. The phased evaluation approach is consistent with its fundamental characteristics, and the nationwide scope of selection results in temporal and spatial variations in 5ATAs evaluations, enabling a "DID" analysis [10]. The consecutive evaluations of 280 scenic areas from 2007 to 2019 provide an ample sample for this study. The paper intends to employ DID analysis to assess the influence of 5ATAs on local tourism economic development. As of 2019, within the 282 prefecture-level cities included in this study, 155 cities had at least one approved 5ATAs. These 155 cities constitute the experimental group, while the remaining cities without 5ATAs form the control group. Following the methodology outlined in Gao and Su [17], a two-way fixed-effects model is applied to implement DID, with the baseline model expressed as:

$$Y_{it}=a_{1}+\beta_{1}{FAS}_{it}+{\alpha_{1}X}_{it}+\gamma_{t}+\mu_{i}+\varepsilon_{it}$$ (1)

Here, *Y*_*it*_ is the dependent variable, where i represents the i-th prefecture-level city and t the t-th year. The study employs four variables to reflect the local tourism economic development level, namely per capita domestic tourism receptions, per capita domestic tourism income, per capita international tourism receptions, and per capita international tourism income [38]. *FAS* is the core explanatory variable, with the estimated coefficient *β*_1_ measuring the net effect of 5ATAs selection on local tourism economic development. *X*_*it*_ denotes control variables, including per capita regional GDP, population density, the development level of the tertiary industry, infrastructure investment, external openness, local general budgetary expenditures, higher education prevalence, public cultural services, healthcare conditions, total passenger volume, and total freight volume [6,17,54]. *γ*_*t*_ and *μ*_*i*_ represent time and region fixed effects, respectively.

### 3.2 Framework

**Fig 1:**
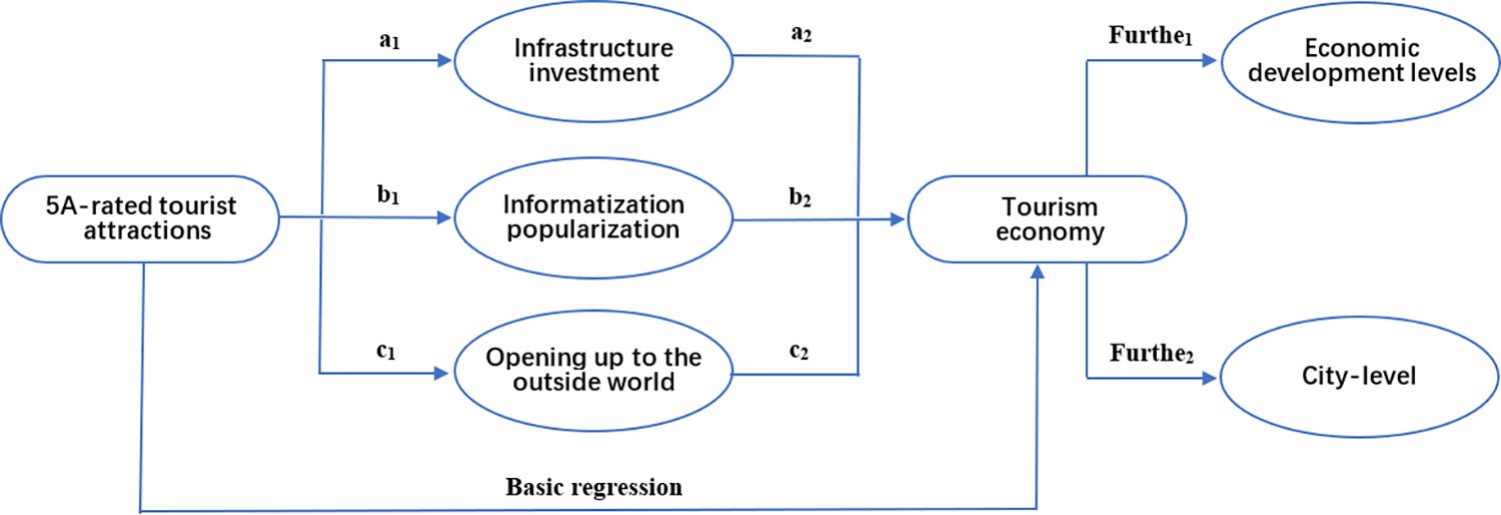
Framework diagram for research.

We have developed a research framework based on the research assumptions and model settings in this article, as shown in Fig 1. To draw an accurate conclusion, this article employs the DID method to explore the impact of 5A selection on the tourism economy. It also employs a series of robustness tests to confirm hypothesis H1. As part of the analysis, a mediation effect model was adopted in order to better understand the underlying mechanism. The intermediary effect analysis steps of this study are illustrated using infrastructure investment in the 5A selection as an example. An examination of whether local tourism has been promoted by the 5A selection is the first step. Tests can be conducted if there is a significant positive impact. The second step consists of testing the impact coefficient a_1_ of 5A selection on infrastructure investment as an intermediary variable. We then test the significance of the coefficient b_1_ of the impact of infrastructure investment on local tourism. When both a_1_ and b_1_ are significant, it indicates that infrastructure investment contributes to the local tourism economy by way of a mediating effect, thus confirming the H2 hypothesis. Moreover, the article examines the differential effect of 5A selection across regions and cities with varying levels of economic development, in order to address hypothesis H3.

### 3.3 Variables and data

This article manually collected data from 299 prefecture-level cities from data sources from 2002 to 2019, covering five years before and thirteen years after policy implementation. There are 31 Chinese provinces represented in the city data, and similar data have been widely used in previous studies [17,26,41,42]. Given that the data from municipalities aligns more closely with provincial administrative levels and is not congruent with prefecture-level cities, it has been omitted from the analysis [17]. Parts of the cities were newly established or disbanded during this period, and some cities had severe data deficiencies. These cities were also excluded to ensure research accuracy [10,17]. This research draws upon 5,076 data entries spanning 282 prefecture-level cities from 2002 to 2019. These entries are derived from authoritative sources including the "China Statistical Yearbook", "China Tourism Yearbook", "China Urban Statistical Yearbook", "China Statistical Yearbook for Regional Economy", "World Bank", "WIND Database", official portals of the Ministry of Culture and Tourism, and the National Bureau of Statistics, as well as provincial and municipal statistical publications [6,10,17,26,41]. We have the relevant permission to use the data. Table 1 provides explanations and calculation methods for the related variables.

1. The Explained Variable: This study aims to assess the influence of 5A selection on the local tourism economy. Drawing upon data acquisition, we have chosen per capita domestic tourist arrivals, per capita domestic tourism income, per capita foreign tourist arrivals, and per capita foreign tourism income as the explained variables [38]. These parameters effectively encapsulate both domestic and international tourist visits and expenditures, thereby offering a holistic gauge of the local tourism economic development. To neutralize the impact of inflation, the study sets 2002 as the base year, calculating consumption indices relative to that year for per capita domestic and international tourism income.
2. Core Explanatory Variables. Based on the list of 5ATAs published by the Ministry of Culture and Tourism, the study constructs the independent variable (*FAS*). In handling this variable, two considerations are made: exclusion of the data from the 22 5ATAs in Beijing, Shanghai, Tianjin, and Chongqing from 2002 to 2019 since the study uses prefecture-level city data; and the consideration that a prefecture-level city may have multiple 5ATAs, thus employing an additive approach to better explore the intensity of the evaluation effects.
3. Control Variables: This study aims to isolate the distinct impact of 5ATAs selection on tourism economic growth by controlling for other influential factors at both economic and social levels. Economically, a region’s overall development can significantly influence its tourism sector. Hence, regional GDP, population density, and the level of tertiary industry development are utilized as indicators of local economic progress. Concurrently, per capita fixed asset investment, the amount of foreign investment utilized per capita, and local government budget expenditures are employed to gauge the tourism economic base and service capacity. Socially, local government initiatives enhancing living standards can affect tourism development. Indicators such as the number of college students per 10,000 people, public library book collections per 100 people, and hospital bed availability reflect cultural and social service levels, and are thus controlled in this analysis. Transportation infrastructure, critical for tourism, is represented by total passenger and freight transport volumes. Moreover, for precise conclusions, we adjust per capita GDP, tertiary industry value added, total fixed asset investment, and local government budget expenditures for inflation using the household price consumption index, with 2002 as the base year.
4. Mediating Variables. Existing research and 5ATAs evaluation criteria guided the selection of infrastructure investment, informatization popularization, and external openness as mediating variables. Infrastructure investment, represented by per capita fixed asset investment, pertains to a city’s and tourism area’s reception capacity, accessibility, and basic service experience, possibly impacting the effect of 5A-level policies. The informatization popularization, represented by the number of internet users per 10,000 people, relates to the promotion of quality signals and local service quality. External openness, represented by the actual amount of foreign investment utilized, is a critical factor in high-quality construction and global development of scenic areas, and may influence tourism economic development promoted by 5ATAs evaluations.

**Table 1:** Explanation and definition of variables.

Variable	Variable interpretation	Variable definitions	Abbreviation
Tour_pcpop_dom	Domestic tourist arrivals per capita	Number of domestic tourists /Total population (Number of people/thousands)	Tppd
Tour_pcinc_dom	Domestic tourism revenue per capita	Domestic tourism revenue /Total population (USD/person)	Tpid
Tour_pcpop_fore	Foreign tourist arrivals per capita	Number of foreign tourists /Total population (Number of people/thousands)	Tppf
Tour_pcinc_fore	Foreign tourism revenue per capita	Foreign tourism revenue /Total population (USD/person)	Tpif
Tour_pcpop_total	Total tourist reception per capita	Total number of tourists /Total population (Number of people/thousands)	Tppt
Tour_pcinc_total	Total tourism income per capita	Total tourism revenue /Total population (USD/person)	Tpit
FAS	Number of 5ATAs	The cumulative number of 5ATAs	FAS
GDPPC	GDP per capita	Actual GDP/total population (Ten thousand dollars per person)	GDPPC
PD	population density	Population per square kilometer (People/km^2^)	PD
PEITTI	The level of development of the tertiary industry	Proportion of employees in the tertiary industry (%)	PEITTI
Infrast_inves	Infrastructure investment	Total investment in fixed assets / Total population (Ten thousand dollars per person)	INF
Opening	Degree of openness to the outside world	The amount of foreign funds actually utilized / Total population (Ten thousand dollars per person)	OPEN
EWTGBOLF	Expenditures within the general budget of local finances	Expenditures within the general budget of local finances (Ten thousand dollars)	EWTGBOLF
Num_uni_stu	Universal access to higher education	Number of university students per 10,000 people	NUS
Pub_libr_coll	Public cultural services	Public library collections per 100 people	PLC
Num_hosp_beds	Medical and health conditions	Number of beds in hospitals and health centers	NHB
Num_theaters	Number of recreational facilities	Number of theaters	NTH
Total_pas_tra	Total passenger traffic	Total passenger traffic (10,000 visitors)	TPT
Tot_vol_shipments	Total volume of shipments	Total volume of shipments (10,000 tons)	TVSH
Inter_popul	The level of informatization development	Number of Internet users / Total population Households/10,000 people)	INT

To acquire a more comprehensive understanding of the data situation across various indicators, this study undertook a descriptive statistical analysis of the dependent variables, core explanatory variables, control variables, and other involved indicators, resulting in Table 2.

**Table 2:** Descriptive statistics.

Variable	Obs	Mean	Std. Dev.	Min	Max
Tppd	4972	5610.878	6822.165	10.72	72537.883
Tpid	4963	831.985	1246.043	0.064	15714.61
Tppf	4964	85.315	301.487	0	4194.872
Tpif	5004	39.422	127.718	0	1627.777
Tppt	4897	5775.5	6924.109	96.872	72698.969
Tpit	4938	875.66	1312.847	0.112	15802.565
FAS	5076	0.333	0.692	0	6
Year	5076	2010.5	5.189	2002	2019
GDP	5076	15332575	21438002	240336	2.693e+08
PD	5022	417.47	310.722	5	2707
Total population	5076	415.109	246.092	15.97	1500
PEITTI	5076	53.908	13.313	4.25	108.03
NTH	5018	15.173	31.207	0	1431
NHB	5076	14832.913	12114.643	865	135812
TPT	5022	8386.076	14206.455	117	365608
NUS	5022	177.063	206.757	2	1294
PLC	5022	53.647	175.032	1	7940
TVSH	5022	11025.549	14649.675	2	554458
CPI	5076	102.473	1.847	97.654	110.087
Province	5076	18.305	7.364	5	31
Exchange rate	5076	7.066	0.744	6.05	8.278
Treat	5076	0.55	0.498	0	1
Level	5076	3.693	1.106	1	5
City	5076	152.539	85.536	2	299
CPIbp	5076	1.277	0.182	1	1.779
GDPPC	5076	3959.952	3401.62	11.683	58396.105
EWTGBOLF	5076	236883.13	289894.82	2972.091	4881626.5
Opening	5020	0.013	0.025	0	0.273
INF	5022	0.044	0.046	0	0.422
INT	5076	1435.604	1684.757	0.041	19865.713

## 4 Results

### 4.1 Basic regression

In accordance with the baseline model, the study employed Stata 17.0 to examine the influence of 5ATAs evaluations on local tourism economics. The regression results can be found in Table 3. As revealed by the data in Table 3, after controlling for time-fixed effects, regional fixed effects, and control variables, the impact of the 5ATAs evaluations on per capita domestic and international tourism receptions and income is significant at the 1% level. As delineated in Table 3, for domestic metrics, 5ATAs selection amplifies the tourism reception by 1700 individuals per thousand and boosts the per capita tourism revenue by 514.7518 US dollars. In terms of international parameters, there’s an enhancement in tourism reception by 32.2498 individuals per thousand, resulting in an increment of per capita tourism revenue by 22.9799 US dollars. The catalytic role of 5ATAs aligns with quality signal theory, serving as labels and guidance within the complex market [9]. Such evaluations have established branding for the selected 5ATAs, attracting both domestic and international tourists, and promoting per capita tourism income from both sectors. As a whole, the evaluation of 5ATAs has a significant positive effect on four tourism economic indicators. Based on this evidence, it can be concluded that 5ATAs has contributed to the development of the local tourism economy. Accordingly, hypothesis H1 supports this viewpoint.

**Table 3:** The impact of 5ATAs selection on tourism economy.

	(1)	(2)	(3)	(4)
	Tppd	Tpid	Tppf	Tpif
FAS	1.7e+03***	514.7518***	32.2498***	22.9799***
	(5.1023)	(7.5481)	(2.8247)	(3.8257)
Controls	Yes	Yes	Yes	Yes
City-fixed effects	Yes	Yes	Yes	Yes
Year-fixed effects	Yes	Yes	Yes	Yes
*Observations*	4972	4963	4964	5004
R-squared	0.7622	0.7470	0.9347	0.8552
F	26.0332	56.9744	7.9788	14.6359

Notes

*, **, *** represent the significance at 10%, 5%, and 1%, respectively.

### 4.2 Robustness test

#### 4.2.1 Parallel trend test

**Fig 2:**
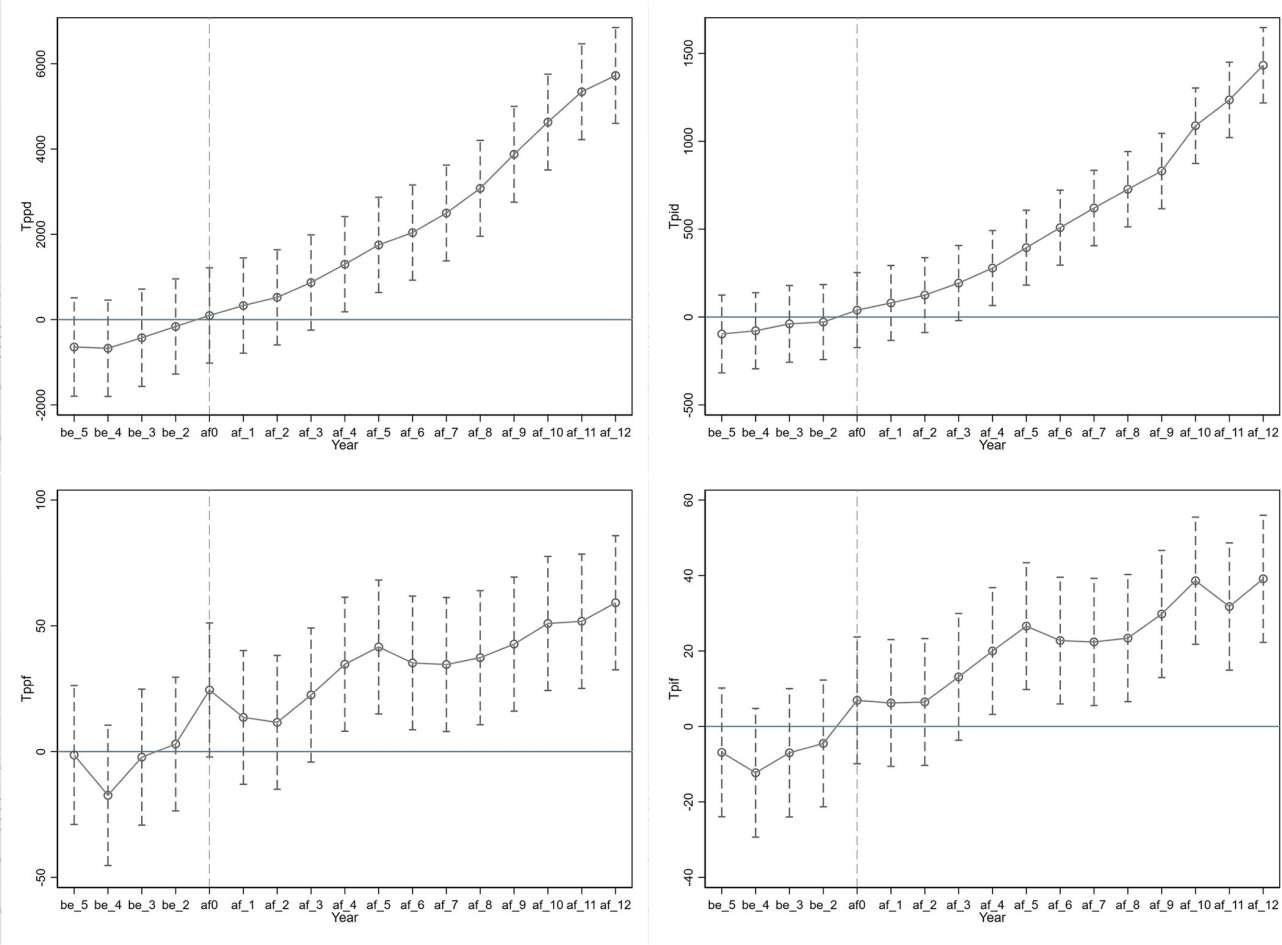
Parallel trend test. Notes: In an endeavor to articulate the developmental trajectory with precision, the notation utilized in this study distinguishes between three temporal phases relative to policy enactment. The symbol ’be’ is assigned to denote the period preceding policy implementation, ’af’ signifies the stage following policy enactment, and ’af0’ is specifically reserved to represent the year of policy execution itself.

A prerequisite for conducting a DID analysis is that there must be no systematic differences between the treatment and control groups prior to the intervention. If the cities in the treatment group were already diverging in development trends compared to the control group before the policy implementation, an accurate assessment of the impact of 5ATAs evaluations on tourism economics would be hindered. This study took the year preceding the policy implementation (2006) as the baseline to test for the parallel trends assumption and dynamic effects within the treatment cities concerning 5ATAs evaluations, as evidenced in Fig 2.

From Fig 2, it is discernible that there was no significant difference in the growth rate of tourism economics between the treatment and control cities before the policy inception, with regression coefficient confidence intervals containing zero. Following a period post-implementation of the 5ATAs evaluations, the regression coefficients were notably positive, manifesting the enhancement of market competitiveness and economic stimulation provided by the quality signals. Within Fig 2, a lag effect of policy promotion can be observed, indicating that a certain period was required for the policy’s efficacy to become pronounced. This delay may have been related to the global financial crisis at the time, as policy promotion effects did not appear instantaneously. Additionally, per capita international tourism receptions and income experienced some fluctuations around 2014. This could have been due to reported issues within the 5ATAs, such as inadequate services, poor quality, and improper charges by managers, all of which may have deterred international visitors and consumption. However, these circumstances began to improve after the introduction of 5ATAs supervision in 2015, revealing substantial potential thereafter.

#### 4.2.2 Placebo test

**Fig 3:**
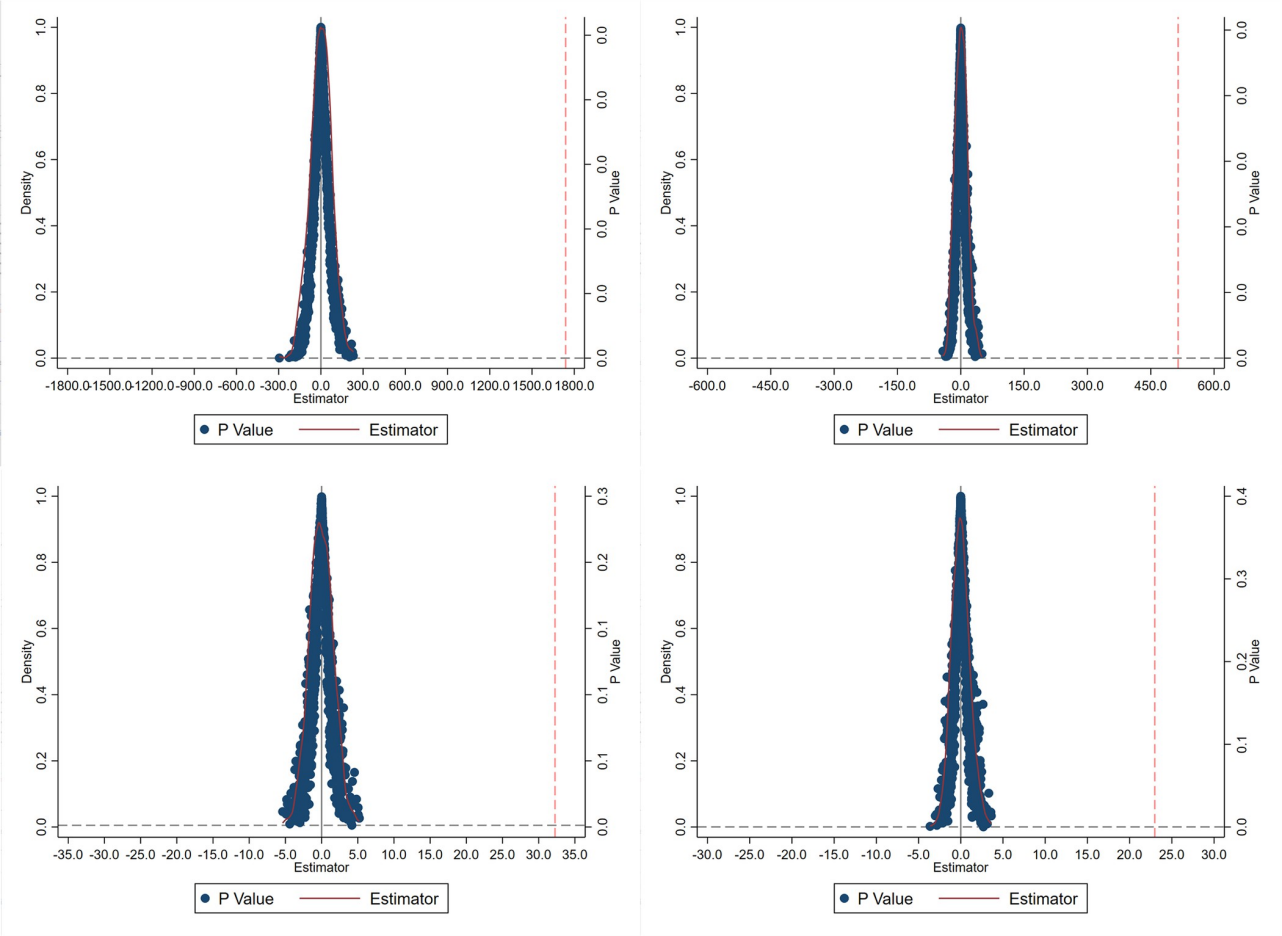
Placebo test.

In this quasi-natural experiment of the current study, the pre-award stage of the 5ATAs for both the treatment and control groups might be subject to the shock and influence of other factors. These could potentially affect the economic benefits generated by the 5A-grade rating, impinge on the assessment of the quality signal effect, and consequently lead to the failure of the hypothesis. Following the approach of Jin and Xi et al.[38], this study rendered the impact of 5ATAs rating on tourism economy as random and conducted regression analyses over 1000 draws, obtaining the resulting coefficients. When the coefficients are concentrated around 0, it can be inferred that the omitted coefficients are zero. Upon examination, as revealed in Fig 3, the randomly processed estimated coefficients follow a normal distribution around 0, indicating that other factors are unlikely to impact the estimation results. This alignment with the expectations of a placebo test enhances the rigor of the causal inference logic in this study, corroborating the robustness of the obtained regression results.

#### 4.2.3 Exclude test results for sub-provincial cities

In the scope of this study, which encompasses 282 cities including 15 sub-provincial cities, the latter are characterized by a higher political status, stronger economic capability, and greater autonomy in urban management. These cities often possess abundant tourism resources and a vast market of potential visitors, but they also face an intensely competitive destination market environment [41]. To derive more precise results, this study opts to exclude the sub-provincial cities and focus on a subsample for regression, the findings of which are presented in Table 4. As discerned from Table 4, the regression results obtained from the subsample, after excluding the sub-provincial cities, remain significantly positive. This means that even when data from sub-provincial cities are omitted, the 5ATAs continue to have a stimulating effect on the development of local tourism economy. This discovery further supports the hypothesis H1 and increases its credibility.

**Table 4:** Exclude test results for sub-provincial cities.

	(1)	(2)	(3)	(4)
	Tppd	Tpid	Tppf	Tpif
FAS	1.7e+03***	476.5844***	29.9743**	18.7552***
	(4.6569)	(6.2237)	(2.2536)	(2.7908)
Controls	Yes	Yes	Yes	Yes
City-fixed effects	Yes	Yes	Yes	Yes
Year-fixed effects	Yes	Yes	Yes	Yes
*Observations*	4705	4698	4694	4734
R-squared	0.7555	0.7228	0.8987	0.8090
F	21.6870	38.7345	5.0787	7.7888

Notes

*, **, *** represent the significance at 10%, 5%, and 1%, respectively.

#### 4.2.4 Test results for replacing the dependent variable

In an effort to observe more directly the comprehensive impact of the 5ATAs selection on local tourism and to explore the role of quality signals in attracting tourists and stimulating economic growth, this study adopts per capita total tourist receptions and per capita total tourism revenue as substitutes for the original indicators [6]. The results are presented in columns (1) and (2) of Table 5. An examination of these columns reveals that the selection of 5ATAs has a significant positive effect on local per capita total tourist receptions and per capita total tourism revenue. Specifically, the selection of areas as 5A-grade has led to an increase in the total number of local tourists and a corresponding growth in total local tourism income. These conclusions are consistent with hypothesis H1.

**Table 5:** Regression results of replacing the dependent variable and shortening the time sample.

	(1)	(2)	(3)	(4)	(5)	(6)
	Tppt	Tpit	Tppd	Tpid	Tppf	Tpif
FAS	1.7e+03***	536.3064***	1.2e+03***	375.6569***	26.9172***	18.6435***
	(5.0114)	(7.4497)	(5.1639)	(7.4562)	(2.7568)	(3.5785)
Controls	Yes	Yes	Yes	Yes	Yes	Yes
City-fixed effects	Yes	Yes	Yes	Yes	Yes	Yes
Year-fixed effects	Yes	Yes	Yes	Yes	Yes	Yes
*Observations*	4897	4938	3349	3346	3355	3357
R-squared	0.7644	0.7567	0.8281	0.8027	0.9627	0.9220
F	25.1145	55.4978	26.6656	55.5942	7.6002	12.8059

Notes

*, **, *** represent the significance at 10%, 5%, and 1%, respectively.

#### 4.2.5 Shorten the test results for time samples

Since 2015, China has undertaken several influential tourism site selection initiatives, such as the National Tourism Resort Selection (begun in 2015), National Comprehensive Tourism Demonstration Area Selection (initiated in 2016), and National Park Selection (started in 2017), among others. To discern the “net effect” of 5ATAs selection on tourism economic growth, it is essential to control for the impact of these other policies [17]. In this study, we constrained the time window to panel data from 2004 to 2015 to carry out regression analyses, thereby excluding the possible influence of other concurrent policies. The outcomes are manifested in columns (3), (4), (5), and (6) of Table 5. On examining the latter four columns of Table 5, it can be discerned that, following the shortening of the time window, the promotional effect of 5ATAs on the tourism economy mirrors that of the baseline regression, consistently displaying a significant positive impact. This conclusion once again proves the validity of hypothesis H1.

#### 4.2.6 Test results after 1% tail off of the dependent variable

In the baseline regression analysis, certain extreme values can influence the results. Within the category of 5ATAs, some destinations are exceptionally well-developed, geographically advantaged, and exhibit tourism visits and economic benefits far surpassing others. Conversely, some 5ATAs fall markedly below others in terms of tourist visits and economic yield for various reasons. Such outliers can potentially skew the evaluation of the 5ATAs’ economic impetus. To mitigate the impact of these extreme values, this study applied a 1% tail-trimming technique to the four dependent variables, and the results are presented in Table 6. According to the outcomes detailed in Table 6, the post-trimmed regression findings align closely with the baseline regression. Both instances underscore that the selection of 5ATAs has a stimulating effect on the local tourism economy, and the impact of quality signaling is pronounced. This discovery further confirms the hypothesis H1.

**Table 6:** The result of the test after being tailed by the explanatory variable.

	(1)	(2)	(3)	(4)
	Tppd_win	Tpid_win	Tppf_win	Tpif_win
FAS	1.7e+03***	474.3123***	31.0244***	21.4592***
	(5.3111)	(8.5445)	(3.0709)	(3.9759)
Controls	Yes	Yes	Yes	Yes
City-fixed effects	Yes	Yes	Yes	Yes
Year-fixed effects	Yes	Yes	Yes	Yes
*Observations*	4972	4963	4964	5004
R-squared	0.7890	0.7969	0.8994	0.8629
F	28.2078	73.0085	9.4307	15.8080

Notes

*, **, *** represent the significance at 10%, 5%, and 1%, respectively.

#### 4.2.7 Test results of added province-time fixed effect

In investigating the interrelation between the two entities, certain latent factors could potentially influence the outcomes. Notably, regional disparities exist in the trajectories of tourism economic development. To mitigate the temporal regional influence, we incorporated the province-time fixed effect into Eq (1), aiming to attenuate biases arising from omitted variables. The regression outcomes are delineated in Table 7. Upon integrating the province-time fixed effect, as evidenced in Table 7, the influence of 5ATAs selection on the local tourism economy remains markedly positive, with statistical significance at the 1% threshold. This robust finding underscores that the 5ATAs selection bolsters the local tourism economy, thereby reaffirming hypothesis H1.

**Table 7:** Test results of added province-time fixed effect.

	(1)	(2)	(3)	(4)
	Tppd	Tpid	Tppf	Tpif
FAS	1.4e+03***	440.8449***	33.8832***	22.0797***
	(14.6351)	(21.9256)	(12.3375)	(13.3370)
Controls	Yes	Yes	Yes	Yes
City-fixed effects	Yes	Yes	Yes	Yes
Year-fixed effects	Yes	Yes	Yes	Yes
Province*Year-fixed effects	Yes	Yes	Yes	Yes
*Observations*	4953	4945	4941	4985
R-squared	0.8622	0.8277	0.9449	0.8879
F	214.1849	480.7321	152.2142	177.8762

Notes

*, **, *** represent the significance at 10%, 5%, and 1%, respectively.

#### 4.2.8 Test results of hysteresis effect

In the context of tourism management, upon achieving a 5A rating, scenic locations often necessitate a transitional period for adequate promotion and preparation, aiming to effectively communicate this elevated quality designation to the broader market. Subsequent to this dissemination of quality assurance, potential visitors typically adopt a cautious approach, leading to a potential temporal delay in the observable economic benefits associated with the 5A tourism accreditation [27]. To quantitatively assess this, we employed regression analysis on Eq (1), incorporating a one-period lag for 5A ratings (denoted as L.FAS), the results of which are presented in Table 8. Notably, the lagged 5A tourism accreditation exhibits a statistically significant positive correlation with the dependent variable at the 1% confidence level. This empirical finding underscores the inherent delay in the economic ramifications of 5A tourism ratings, corroborating the observations delineated in Fig 2 and bolstering the veracity of Hypothesis H1.

**Table 8:** Test results of hysteresis effect.

	(1)	(2)	(3)	(4)
	Tppd	Tpid	Tppf	Tpif
L.FAS	1.8e+03***	535.4578***	31.5028**	23.7460***
	(4.6714)	(7.1196)	(2.5687)	(3.8430)
Controls	Yes	Yes	Yes	Yes
City-fixed effects	Yes	Yes	Yes	Yes
Year-fixed effects	Yes	Yes	Yes	Yes
*Observations*	4722	4717	4713	4736
R-squared	0.7683	0.7559	0.9386	0.8628
F	21.8220	50.6882	6.5984	14.7683

Notes

*, **, *** represent the significance at 10%, 5%, and 1%, respectively.

#### 4.2.9 Test results of increment control variable

In recent times, China’s government has actively pursued the enhancement and transformation of its industrial structure. This shift in industrial dynamics could potentially impact tourism economics, presenting both novel opportunities and challenges. Local residents’ savings, indicative of financial development, may also influence tourism economic growth. To more precisely determine the net influence of 5ATAs selection on tourism economics, this study, drawing on prior research, introduces two additional metrics—industrial structure and household savings—as control variables [10,40,48]. The industrial structure is quantified by the ratio of tertiary industry value-added to GDP, while household savings are gauged by the proportion of urban and rural residents’ total year-end savings to GDP. Utilizing Eq (1), these indicators are integrated as control variables. The results, presented in Table 9, reveal that the inclusion of these controls confirms a significant effect of 5A selection on tourism economics at the 1% level, aligning with the foundational regression outcomes. This finding denotes a substantial positive impact of 5ATAs selection on tourism economic development, demonstrating robustness and strongly supporting hypothesis H1.

**Table 9:** Test results of increment control variable.

	(1)	(2)	(3)	(4)
	Tppd	Tpid	Tppf	Tpif
FAS	1.7e+03***	514.6021***	32.2431***	22.9756***
	(5.1005)	(7.5462)	(2.8240)	(3.8247)
Controls	Yes	Yes	Yes	Yes
City-fixed effects	Yes	Yes	Yes	Yes
Year-fixed effects	Yes	Yes	Yes	Yes
*Observations*	4971	4962	4963	5003
R-squared	0.7622	0.7470	0.9347	0.8552
F	26.0150	56.9451	7.9749	14.6286

Notes

*, **, *** represent the significance at 10%, 5%, and 1%, respectively.

### 4.3 Mechanism test

#### 4.3.1 The effect of 5ATAs selection on mediation variables

In the review of the existing literature, we identified that investments in infrastructure can enhance local transportation, augment reception capacity, and improve the quality of public services. The level of information technology adoption is a crucial criterion in the construction of 5ATAs and represents an essential means for tourists to access information about these locations. Moreover, it serves as a vital pathway to elevate the quality of tourism services. The external openness symbolizes a city’s international development and attractiveness and is vital for transmitting quality signals to international markets and attracting foreign tourists. Collectively, these three factors play unique roles in local tourism development. This study referenced the practices of other scholars and constructed a mediating effects model to validate hypothesis H2 [55].

This study aims to analyze whether investments in infrastructure, the informatization popularization, and the external openness act as mediators in the influence of 5ATAs selection on the tourism economy. The analysis was conducted in three stages, culminating in the formulation of Eq (2) and Eq (3) [28,55]:

$$Z_{it}=a_{2}+\beta_{2}{FAS}_{it}+\alpha_{2}X_{it}+\gamma_{t}+\mu_{i}+\varepsilon_{it}$$ (2)

$$Y_{it}=a_{3}+\beta_{3}Z_{it}+\beta_{4}{FAS}_{it}+\alpha_{3}X_{it}+\gamma_{t}+\mu_{i}+\varepsilon_{it}$$ (3)

Here, *Z* represents the mediator variables, namely investments in infrastructure, informatization popularization, and the external openness, while the meanings of other variables remain unchanged. To explore whether the selection of 5ATAs has an impact on these mediators, according to Eq (2), the selection of 5ATAs was employed as the independent variable, and the mediator variables were used as dependent variables. The regression results are summarized in Table 10. Upon examination of Table 10, it becomes evident that the coefficients corresponding to investments in infrastructure and informatization popularization in relation to 5ATAs selection are significantly positive. This indicates a notable enhancement in infrastructure investments, informatization popularization, and external openness influences due to the selection of 5ATAs.

**Table 10:** The effect of 5ATAs selection on mediation variables.

	(1)	(2)	(3)
	INF	INT	OPEN
FAS	0.0123***	278.3647***	0.0036***
	(5.5486)	(4.0194)	(2.6741)
Controls	Yes	Yes	Yes
City-fixed effects	Yes	Yes	Yes
Year-fixed effects	Yes	Yes	Yes
*Observations*	5022	5076	5020
R-squared	0.7847	0.7926	0.8005
F	30.7875	16.1555	7.1510

Notes

*, **, *** represent the significance at 10%, 5%, and 1%, respectively.

#### 4.3.2 The impact of infrastructure investment on the tourism economy

According to Eq (3), utilizing investments in infrastructure as the independent variable, and employing four metrics representing tourism economy as dependent variables, the regression results were obtained and are presented in Table 11. Within Table 11, the regression results show a significantly positive relationship between infrastructure investments and tourism economy, denoting that infrastructure investment indeed fosters tourism economic growth. In conjunction with the findings from Table 10 and the mediation effect model, it can be affirmed that the selection of 5ATAs indeed stimulates local tourism economic development by increasing infrastructure investment. Specifically, investments in infrastructure serve as a mediator in the facilitation of tourism economy by the 5ATAs selection, Supporting the content of hypothesis H2.

**Table 11:** The impact of infrastructure investment on the tourism economy.

	(1)	(2)	(3)	(4)
	Tppd	Tpid	Tppf	Tpif
INF	3.8e+04***	1.0e+04***	305.2474**	307.9986***
	(3.4098)	(3.9606)	(2.0411)	(2.6446)
FAS	1.3e+03***	387.1803***	28.6168**	19.1099***
	(3.6568)	(5.7228)	(2.3247)	(3.1439)
Controls	Yes	Yes	Yes	Yes
City-fixed effects	Yes	Yes	Yes	Yes
Year-fixed effects	Yes	Yes	Yes	Yes
*Observations*	4927	4918	4919	4959
R-squared	0.7771	0.7792	0.9353	0.8579
F	17.9869	39.4173	9.7966	9.8681

Notes

*, **, *** represent the significance at 10%, 5%, and 1%, respectively.

#### 4.3.3 The impact of informatization popularization on tourism economy

In line with Eq (3), the informatization popularization was incorporated into the regression analysis as the explanatory variable, with four metrics symbolizing the tourism economy serving as the dependent variables. The regression outcomes, as detailed in Table 12, reveal a significantly positive association between the informatization popularization and tourism economics. In synthesis, the selection of 5ATAs promotes local tourism economic development by enhancing the dissemination of high-quality information, augmenting visibility, expanding influence, and consequently transmitting premium quality signals. Specifically, the informatization popularization acts as a mediating factor in the advancement of tourism economy through the 5ATAs selection, thus corroborating hypothesis H2.

**Table 12:** The impact of informatization popularization on tourism economy.

	(1)	(2)	(3)	(4)
	Tppd	Tpid	Tppf	Tpif
INT	0.8247***	0.2208***	0.0133***	0.0132***
	(4.1276)	(5.3541)	(2.7230)	(3.2140)
FAS	1.5e+03***	454.2836***	28.6104**	19.3505***
	(4.3895)	(7.0704)	(2.4357)	(3.2859)
Controls	Yes	Yes	Yes	Yes
City-fixed effects	Yes	Yes	Yes	Yes
Year-fixed effects	Yes	Yes	Yes	Yes
*Observations*	4972	4963	4964	5004
R-squared	0.7707	0.7653	0.9359	0.8615
F	19.6238	40.2677	8.2704	12.7270

Notes

*, **, *** represent the significance at 10%, 5%, and 1%, respectively.

#### 4.3.4 The impact of external openness on the tourism economy

In alignment with Eq (3), the study employed the external openness as the explanatory variable, considering four representative metrics of tourism economy as the dependent variables. The corresponding regression results, as detailed in Table 13, reveal a pronounced positive effect of external openness on the tourism economy, indicating its role in promoting growth within this sector. This finding underscores the effect of the Chinese government’s ongoing initiatives to widen its external openness, particularly through the attraction of foreign investment. The infusion of foreign capital has led to both the refinement of services and an uplift in the quality of scenic areas, furthering the dissemination of quality signals on a broader scale and subsequently enhancing the development of the tourism economy. In sum, the degree of external openness has acted as an intermediary in the encouragement of the tourism economy by the selection process for 5ATAs. This conclusion offers robust support for hypothesis H2 of the study, highlighting the pivotal role of external openness in the intricate dynamics of tourism economics.

**Table 13:** The impact of external openness on the tourism economy.

	(1)	(2)	(3)	(4)
	Tppd	Tpid	Tppf	Tpif
OPEN	4.2e+04***	9.6e+03***	1.1e+03***	555.6108***
	(3.4614)	(3.9738)	(3.5833)	(2.9168)
FAS	1.6e+03***	483.2078***	28.6269**	20.9824***
	(4.9709)	(7.1793)	(2.4631)	(3.2712)
Controls	Yes	Yes	Yes	Yes
City-fixed effects	Yes	Yes	Yes	Yes
Year-fixed effects	Yes	Yes	Yes	Yes
*Observations*	4927	4918	4919	4959
R-squared	0.7676	0.7550	0.9364	0.8577
F	18.3934	39.7239	13.4963	16.8301

Notes

*, **, *** represent the significance at 10%, 5%, and 1%, respectively.

### 4.4 Testing for heterogeneity

#### 4.4.1 Test for heterogeneity of economic development level

Though the selection of 5ATAs has been demonstrated to promote local tourism economic growth, its effects might exhibit heterogeneity. Regional factors often play a significant role [56], and given China’s vast territory, substantial disparities exist in economic development levels across different areas. The investment in and attention to the tourism industry may vary, and consequently, the effects of quality signals from 5ATAs selection might differ. The eastern coastal regions of China offer abundant development opportunities, concentration of resources, and higher levels of economic growth. In contrast, central and western regions lag in development due to geographical factors. To explore the impact of 5ATAs selection policy on regions with varying economic development levels, the study categorizes Chinese provinces into eastern, central, and western divisions [41], as enumerated in Table 14. Subsequent analyses delve into the influence of 5ATAs on the tourism economy across these three regions of disparate economic advancement, with the results detailed in Tables 15–17.

**Table 14:** Regional division.

Regional	Including provinces
Eastern region	Hebei Province, Liaoning Province, Jiangsu Province, Zhejiang Province, Fujian Province, Shandong Province, Guangdong Province, Hainan Province
Central region	Shanxi Province, Jilin Province, Heilongjiang Province, Anhui Province, Jiangxi Province, Henan Province, Hubei Province, Hunan Province
Western region	Sichuan Province, Guizhou Province, Yunnan Province, Guangxi Zhuang Autonomous Region, Inner Mongolia Autonomous Region, Tibet Autonomous Region, Shaanxi Province, Gansu Province, Qinghai Province, Ningxia Hui Autonomous Region, Xinjiang Uygur Autonomous Region

**Table 15:** The impact of 5ATAs selection on the tourism economy in the eastern region.

	(1)	(2)	(3)	(4)
	Tppd	Tpid	Tppf	Tpif
FAS	1.3e+03***	518.3982***	12.0388	18.1333*
	(3.2404)	(5.1732)	(1.0593)	(1.6688)
Controls	Yes	Yes	Yes	Yes
City-fixed effects	Yes	Yes	Yes	Yes
Year-fixed effects	Yes	Yes	Yes	Yes
*Observations*	1716	1714	1744	1746
R-squared	0.7807	0.7604	0.9533	0.8657
F	10.5002	26.7620	1.1222	2.7851

Notes

*, **, *** represent the significance at 10%, 5%, and 1%, respectively.

**Table 16:** The impact of 5ATAs selection on the tourism economy in the central region.

	(1)	(2)	(3)	(4)
	Tppd	Tpid	Tppf	Tpif
FAS	2.3e+03***	476.1098***	51.2794*	20.5216**
	(3.2170)	(3.8793)	(1.7653)	(2.0919)
Controls	Yes	Yes	Yes	Yes
City-fixed effects	Yes	Yes	Yes	Yes
Year-fixed effects	Yes	Yes	Yes	Yes
*Observations*	1704	1703	1699	1704
R-squared	0.7659	0.7450	0.7316	0.6672
F	10.3489	15.0488	3.1162	4.3760

Notes

*, **, *** represent the significance at 10%, 5%, and 1%, respectively.

**Table 17:** The impact of 5ATAs selection on the tourism economy in the western region.

	(1)	(2)	(3)	(4)
	Tppd	Tpid	Tppf	Tpif
FAS	2.3e+03***	554.9805***	40.1175**	25.2053***
	(3.3285)	(3.7279)	(2.3667)	(3.0931)
Controls	Yes	Yes	Yes	Yes
City-fixed effects	Yes	Yes	Yes	Yes
Year-fixed effects	Yes	Yes	Yes	Yes
*Observations*	1552	1546	1521	1554
R-squared	0.7689	0.7369	0.7696	0.7437
F	11.0789	13.8972	5.6013	9.5673

Notes

*, **, *** represent the significance at 10%, 5%, and 1%, respectively.

Inspection of Table 15 reveals that the selection of 5ATAs has exerted a significant positive influence at the 1% level on per capita domestic tourist arrivals and income in eastern regions. However, this effect does not extend to a significant positive impact on per capita foreign tourist arrivals, while the influence on per capita foreign tourism income is significant at the 10% level. These findings indicate that the selection of 5ATAs promotes domestic visitation and tourism consumption but plays an inconspicuous role in attracting foreign tourists and does not markedly affect foreign tourism revenue. One plausible explanation for this observation may be the existing frequency of international interactions and communications in eastern regions, coupled with an abundance of urban tourism and other scenic resources. With fierce competition among tourism destinations and diverse travel choices available to international tourists, the selection of a site as a 5ATAs may not provide significant assistance in attracting foreign visitors or notably enhance foreign tourism economy. This nuanced understanding emphasizes the complex interactions between domestic and international tourism dynamics in regions with varying levels of global engagement. It provides a compelling insight into the multifaceted nature of tourism promotion and the limitations of certain quality signaling strategies in a highly competitive and diverse tourism landscape.

An examination of Table 16 reveals that the selection of 5ATAs has had a significant positive impact on per capita domestic and foreign tourist arrivals and income in central regions. The effect is particularly pronounced in attracting domestic tourists and spurring the growth of domestic tourism economy. For the central regions, where international tourism is not highly developed and the population is relatively dense, the inclusion in the 5ATAs allows the destination to quickly stand out in the tourism market. This selection serves as a reference and recommendation for both domestic and foreign tourists’ destination choices, thus promoting international tourism development to a substantial degree. These observations in central regions underscore the influential role of quality signaling in the development of both domestic and international tourism. They further illuminate the distinct characteristics of tourism dynamics in different regions, emphasizing how strategic positioning and quality recognition can leverage regional strengths to foster tourism growth in a nationally and globally competitive marketplace.

An analysis of Table 17 unveils that the selection of 5ATAs has led to a marked positive effect on per capita domestic and international tourism receptions and income in the western regions, with the degree of enhancement even surpassing that of the central regions. Upon attaining the 5A status, scenic areas in the western regions experience an increase in visibility. Capitalizing on their distinctive geographical landscapes and ethnic charm, these areas have succeeded in attracting a substantial number of tourists, thereby efficaciously augmenting both domestic and international visitor numbers and tourism revenue. This finding highlights the potency of the 5A-grade rating system in harnessing the unique attributes of the western regions, demonstrating its capability in stimulating tourism and contributing to economic growth. Moreover, it underlines the role of quality accreditation in diversifying and enhancing the appeal of a region, drawing attention to the importance of leveraging intrinsic qualities to foster a sustainable tourism industry in varying regional contexts.

By synthesizing the data from Tables 15–17, a conclusive observation can be drawn that the 5ATAs selection has yielded disparate impacts across the eastern, central, and western regions. Specifically, the effects of the 5ATAs selection on the tourism economy vary according to the levels of economic development in different regions. This may be because the eastern region has a good economic foundation, rich resources, complete tourism facilities, and the selection activities have limited driving effect on the development of the tourism economy in the eastern region. The economic development level of the central and western regions is relatively backward, and the selection results can bring brand effect, attract more tourists to visit, and better drive the local tourism economy. This finding offers empirical support for hypothesis H3, positing that the stimulative role of the 5ATAs selection manifests differentiated performances across regions with distinct economic development stages. The study thus highlights the nuanced interplay between tourism policy instruments, such as quality accreditation, and regional economic contexts, contributing to our understanding of how spatial variations in economic conditions may mediate the efficacy of tourism development strategies.

#### 4.4.2 Examination of city-level heterogeneity

China’s diverse array of cities, varying widely in their developmental stages, are categorized into different grades based on criteria such as political standing, economic prowess, urban scale, regional influence, and population size. In alignment with the 2022 rankings by the China Business Network Research Institute (CBNRI), this study employs existing data to segment cities into groups. Forty-five cities, excluding the four municipalities of Beijing, Shanghai, Tianjin, and Chongqing, are defined as high-level cities, encompassing first-tier, new first-tier, and second-tier cities. Conversely, 237 cities are classified as low-level, spanning third, fourth, and fifth-tier cities. An examination of the heterogeneous impact of 5ATAs selection on the tourism economy of cities with different scales is encapsulated in Table 18. An inspection of Table 18 reveals a distinct heterogeneity in the influence of 5ATAs selection across various city grades. A significant positive effect is discerned on the tourism economy of low-level cities, whereas the positive impact on high-level cities is confined to per capita domestic tourism revenue. After being rated 5A grade assessment, with the quality signal effect, it can form a competitive advantage in the complex and diverse tourism market, increase the number of tourists and tourism revenue by expanding popularity and forming a brand, while high-level cities have stronger strength to build the brand image of tourist attractions, and already have a good tourism foundation, and the evaluation of 5A scenic spots cannot bring all-round improvement. These results elucidate the differentiated impact of the 5ATAs selection across city grades and further buttress hypothesis H3. This contributes to a nuanced understanding of spatial differentiation in the influence of tourism policies, underscoring the necessity of a tailored approach considering the unique characteristics and developmental stages of individual urban areas.

**Table 18:** Examination of city-level heterogeneity.

	HIGH				LOW			
	(1)	(2)	(3)	(4)	(5)	(6)	(7)	(8)
	Tppd	Tpid	Tppf	Tpif	Tppd	Tpid	Tppf	Tpif
FAS	682.9999	300.3414***	-0.1836	1.0441	2.2e+03***	498.4582***	41.1684**	20.1037**
	(1.4096)	(3.7171)	(-0.0137)	(0.0638)	(4.7621)	(4.7141)	(2.2356)	(2.2383)
Controls	Yes	Yes	Yes	Yes	Yes	Yes	Yes	Yes
City-fixed effects	Yes	Yes	Yes	Yes	Yes	Yes	Yes	Yes
Year-fixed effects	Yes	Yes	Yes	Yes	Yes	Yes	Yes	Yes
*Observations*	801	798	810	810	4171	4165	4154	4194
R-squared	0.8003	0.8577	0.9582	0.8843	0.7500	0.7019	0.7517	0.6716
F	1.9871	13.8165	0.0002	0.0041	22.6777	22.2223	4.9980	5.0098

Notes

*, **, *** represent the significance at 10%, 5%, and 1%, respectively.

## 5 Discussion

### 5.1 Influence of 5ATAs selection on the tourism economy

Prior investigations have illuminated the capacity of 5ATAs selection to stimulate the local tourism sector, augmenting both local tourism influx and per capita tourism revenue. Specifically, the local scenic area was rated 5ATAs corresponds to an enhancement in per capita domestic tourism by over 1,700 individuals per thousand, a rise in local per capita domestic tourism revenue by 514.7518 US dollars, an increase in per capita international tourism by 32.2498 individuals per thousand, and a surge in per capita international tourism revenue by 22.9799 US dollars. Robustness checks further substantiate the veracity and significance of these observations, affirming hypothesis H1. Some scholars have previously examined the effect of 5ATAs establishment on the local economy in their research, concluding that 5A establishment can encourage the growth of local GDP [6]. Earlier research has examined scenic spot selection at the 3A level, indicating that such selection positively influences the attraction of international tourists [19]. The findings of this study corroborate these earlier conclusions. In exploring the impact of 5A selection on local tourism economy, this study employs quality signal theory, alongside the DID method, to yield more precise outcomes. The results demonstrate that 5A selection substantially fosters the development of local tourism economy and contributes to the enrichment of related research.

A world-class scenic spot with strong brand appeal, 5A is China’s highest level of tourist attractions. Among the many industry events taking place each year, the 5ATAs selection is one of the most anticipated and serves as a strong quality signal in the vast tourism market. In addition to enhancing their visibility and increasing their influence, the 5A selection will also force local residents to continuously improve their own construction, thereby increasing the number of tourists visiting and playing, thereby increasing the income of local residents from tourism. By the end of 2018, 5ATAs, constituting less than 1% of China’s over 30,000 scenic spots, remarkably contributed to 50% of the country’s tourism volume and 70% of its tourism revenue [6]. The selection of 5ATAs, characterized by rigorous standards and substantial influence, occupies a significant market share in China’s tourism sector. Furthermore, this selection process invigorates the tourism market, bolsters its prosperity, and catalyzes regional economic growth in tourism. As with any domestic tourism development, the number of domestic tourists and the income from domestic tourism are much higher than the number of foreign tourists and the income from international tourism, which is also in line with China’s current reality. The majority of tourists coming to China come from within the country, which may be due to China’s large population base and the need for some regions to improve their internationalization level.

### 5.2 Mechanisms underpinning the influence of 5ATAs selection on tourism economic expansion

It is demonstrated through three steps in the mechanism testing section of the article that infrastructure investment, informatization popularization, and external openness play a mediating role in the impact of 5ATAs selection on the tourism economy, thereby supporting hypothesis H2. Prior research has identified the critical role of infrastructure investment in analyzing the influence of the Western Development Strategy on the tourism economy [26]. The widespread adoption of informatization [52] and the degree of regional external engagement [36] have been empirically established as key determinants in the development of the tourism economy. These findings highlight the significant impact of infrastructure investment, informatization adoption, and external openness on the trajectory of tourism economic growth. This study further reinforces the importance of these factors.

Infrastructure investment stands as a paramount indicator of urban amenities and conditions conducive to tourism. Enhanced infrastructure invariably correlates with an elevated visitor experience. Greater investment in this domain not only augments the quality of urban infrastructure but also underscores the significance of 5ATAs, catalyzing governmental focus toward tourist sites. Such attention often translates to increased state-sponsored infrastructure development, culminating in an enriched experience for tourists. Optimal support facilities invigorate the tourists’ inclination to explore and expand, thereby amplifying visitor numbers and augmenting local revenue streams. Research conducted by other academics has demonstrated that the Western development strategy enhances tourism development through infrastructure improvements. Additionally, it has been established that infrastructure investment positively influences economic growth [1,26]. The empirical results of this paper further confirm that governments can promote the tourism economy through the increasing of infrastructure investment.

Over the past few years, information technology has attracted a great deal of attention. Online publicity about scenic spots was relatively limited during the 5ATAs selection period, and network technology was not widely used. 5ATAs selection is a government-organized selection activity with a high level of reliability and guidance and is an excellent quality indicator. A high level of informatization is an important factor in the selection of scenic spots for the 5ATAs, and the government will promote the popularization of local informatization vigorously in order to assist the selection process. Local informatization popularization plays an important role in improving the quality of tourist services in scenic areas. With stronger popularization rates of informatization, local residents are more likely to become aware of such consultations and spread them after on-site visits, contributing to the development of 5ATAs as a brand and its expansion [57]. This contributes to the scenic area’s market penetration, attracting a larger visitor base and enhancing consumption of local offerings. Essentially, due to the significant impact of 5ATAs selection criteria on service quality and informatization popularization, the selection process fosters the widespread adoption of informatization locally and elevates information standards through 5ATAs’ extensive societal influence. In turn, greater informatization visibility amplifies 5ATAs’ appeal, thereby fostering local tourism economic growth. As a result, the informatization popularization has significantly contributed to the development of the local tourism economy within the 5ATAs.

In evaluating the international advancement of regional economy, external openness emerges as a crucial metric, reflecting interactions and economic exchanges with the global community. Research has established that external openness not only augments local tourist influx [36] but also stimulates economic development [1]. Our investigation reveals that the selection of the 5ATAs significantly augments the visibility of the designated scenic regions, thereby amplifying both domestic and global outreach. Elevated standards of openness, coupled with superior service quality, have culminated in a surge of tourist influx and expenditure, subsequently elevating the tourism revenue for local inhabitants. Consequently, 5ATAs selection promote tourism economy through the external openness.

### 5.3 Heterogeneity of 5ATAs on tourism economy

According to this study, the 5ATAs selection has varying degrees of influence on tourism economic development in regions with different levels of economic development. In addition, different cities have differing effects on the tourism economy. Based on the evidence presented, hypothesis H3 is supported. Consistent with prior research findings, this study reveals that policies perform unevenly in regions with differing levels of economic development and in cities with different levels [10,17,38,55]. This insight is instrumental in enhancing the efficacy of the selection policy.

China, characterized by its expansive geographical expanse, exhibits pronounced regional disparities and developmental heterogeneity. The eastern provinces, distinguished by their relatively planar topography, have historically been conduits of international engagement, underpinned by a robust economic infrastructure, an abundance of coastal ports, and seamless integration with global markets. This region, marked by its vibrant commercial undertakings, boasts a formidable economic prowess and a comprehensive suite of service amenities. Upon achieving a 5A rating, these locales effectively galvanize domestic tourism and consumption. Yet, the region’s extensive external interactions and a saturated competitive landscape curtail its ability to allure additional international visitors, rendering only marginal enhancements in the international tourism revenue for local inhabitants.

Conversely, the central and western provinces, with their intricate terrains, confront certain transportation impediments. Their economic bedrock is comparatively tenuous, and their global openness quotient lags behind their eastern counterparts. Service infrastructures in these regions warrant further refinement. Post-certification, the scenic vistas in these provinces distinguish themselves amidst a less crowded competitive arena, garnering widespread acclaim. Endowed with pristine tourism assets and augmented visibility, these regions magnetize a broader tourist demographic, bolstering both domestic and international tourist reception metrics and amplifying per capita tourism revenue streams.

Based on the CBNRI ranking, this study categorizes cities into high-level and low-level cities. It was found that 5ATAs selection has limited effects on attracting tourists and increasing the income of residents in high-level cities, whereas it has a larger impact on attracting tourists and increasing the income of residents in low-level cities. This may be attributed to the fact that high-level cities have a higher level of development, a greater number of tourist attractions, and an overall high level of service. There are a variety of places in which tourists can play and consume in accordance with their interests. A scenic area that has been selected for 5ATAs cannot fundamentally improve its competitiveness and attractiveness, and the degree of advancement is limited. Low-level cities, however, do not have strong foundations, the tourism consumption market still needs to be improved, and there are few high-quality tourism destinations. Upon distinguishing themselves through the 5ATAs selection, scenic locales garner immediate societal attention. This distinction serves as a potent indicator of quality within the tourism sector, bolstering brand prominence. Consequently, these locales establish a pronounced competitive edge, leading to enhanced tourist attraction metrics and a subsequent augmentation in the tourism revenue for local residents.

These insights inform the implementation strategy for 5ATAs selection, enhancing policy efficacy to better regulate the market and foster development. This phenomenon is not limited to 5ATAs selection; similar challenges are prevalent in other selection initiatives. Hence, both governments and organizers should consider varying development levels and adopt more tailored policy approaches. Additionally, the experiences from China’s 5ATAs selection could offer partial guidance for analogous policies in other nations.

## 6 Conclusion and policy recommendations

### 6.1 Conclusion

Since the advent of the new millennium, coinciding with China’s burgeoning economic and societal advancement, tourism has metamorphosed into a way of life, inspiring an increasing populace to venture beyond their homes to relish the nation’s scenic splendor and profound cultural heritage. China’s openness has showcased its rich culture and majestic landscapes, drawing numerous domestic and international visitors. The selection of 5ATAs has established benchmarks for a vast tourism market, branding China’s world-class sites. This evaluation has become a target and direction for the construction of tourist destinations, and studying the effectiveness of the 5ATAs selection policy is vital, although further contributions to this field are needed.

In this paper, the selection of 5ATAs is viewed as a quasi-natural experiment. Utilizing a two-way fixed-effects model and drawing on quality signaling theory, the study employs data from 282 prefecture-level cities in China from 2002 to 2019. After controlling for local economic development levels, local tourism reception capability and service scale, urban cultural standards, and local transportation conditions, the investigation probes the policy’s influence on four key indicators: per capita domestic tourism receptions, per capita domestic tourism revenue, per capita international tourism receptions, and per capita international tourism revenue. Following parallel trend and dynamic effect tests, exclusion of sub-provincial cities, replacement of dependent variables, shortening of the time sample, placebo tests, and tail-trimming treatment of dependent variables, the results remain robust. Mechanism tests elucidate the influential factors and pathways at play. The paper also conducts a series of heterogeneity tests on the conclusion, exploring the effects of the policy on the tourism economy across different regions and city grades.

The results demonstrate that 5ATAs selection has catalyzed local tourism economic development, corroborating the study’s hypothesis H1. A comprehensive suite of robustness analyses substantiates this assertion, signifying that the 5ATAs selection’s quality signal adeptly directs tourists’ destination predilections. Investments in infrastructure have augmented both the caliber of services and the capacity for reception. Concurrently, enhanced local information dissemination has amplified the reach of these quality signals, enriching the overall travel experience. Enhanced levels of openness have precipitated a pronounced increase in both tourist arrivals and expenditure, thereby amplifying the tourism-derived revenue for the local populace. Empirical data further underscores the intermediary roles of infrastructure investment, informatization popularization, and external openness. These insights robustly endorse hypothesis H2. Additionally, the research identifies that in the nascent central and western regions, characterized by a fledgling and less competitive tourism market, the potent quality signal has markedly augmented tourist allure and economic invigoration. In the context of city classification, low-level cities with 5ATAs, through brand consolidation and quality validation, have adeptly leveraged the advantages of 5A-grade categorization. These inferences bolster hypothesis H3.

### 6.2 Policy recommendations

From the foregoing conclusions, the following insights can be gleaned:

The Importance of Quality Signal Theory in Tourism: Historically, quality signal theory has been predominantly deployed in sectors such as healthcare, food, and finance. The present study extends this theoretical framework to the domain of tourism, where quality signals manifest a potent efficacy in promoting destinations and attracting visitors. In the context of asymmetric information in the tourism market, quality signals delineate quality tiers by revealing quality through third-party authoritative labeling, conferring advantages in competitive scenarios. This is also of paramount significance to market management, innovative development of scenic areas, and the overall image of the tourism industry. Governments can further engage in authoritative rating activities, encouraging advancement and stimulating market vitality and competition. Furthermore, quality signals can be expanded to include tourism services, products, and organizations, furnishing theoretical support and fresh impetus for the high-quality development of the tourism industry.

Infrastructure Optimization, informatization popularization, and Reform in 5ATAs: Previous studies have substantiated the essential role of infrastructure in the growth of tourism. The empirical analysis herein further verifies the mediating effect of infrastructure investment and illustrates the mediating role of informatization popularization and external openness on tourism economy. Selection for 5A-grade spurs local governments and scenic areas to augment investment in infrastructure, culminating in improved transportation, better facilities, and superior services to allure more tourists. Informatization popularization is equally vital, with internet penetration among local residents enhancing quality signals and local brand promotion. Our conclusions clearly highlight the key factors that can help boost the local tourism economy. Local government agencies must develop development strategies that focus on these factors. This requires increased investment in infrastructure and the use of digital platforms, particularly social media, to spread the word, while increasing openness and increasing reach.

Policy Implications of 5ATAs Selection: Our findings underscore that selection policies exhibit heightened efficacy and drive substantial economic momentum in regions that are less developed and in cities of a lower tier. Such policies can potentially bridge economic disparities, fostering inclusive prosperity. It is recommended that national cultural and tourism entities extend preferential policies and augmented support to these areas, including increased quotas. Concurrently, it is incumbent upon less developed regions and low-level cities to optimize their selection methodologies. By enhancing quality through meticulous selection processes and broadcasting robust quality indicators, they can carve out distinctive brand niches, thereby catalyzing tourism economic growth. These findings not only guide future 5ATAs selection towards more effectively stimulating the tourism economy but also offer insights for other domestic and international selection policies. Prior to initiating other selections, comprehensive market analysis and scientific strategizing are imperative. Concurrently, it is crucial to enhance protections, upgrade tourism services through selection criteria, and thereby advance the tourism economy. Lastly, regulatory bodies should intensify supervision, refine entry and exit protocols, manage dynamically in real-time, and safeguard the integrity and public perception of certifications.

### 6.3 The prospect of research

Certainly, the present study exhibits some limitations, thereby providing avenues for future refinement and exploration. (1) Quality Signal Theory. While replete with rich content, quality signal theory offers vast space for application within the tourism domain. Future research can further enrich and expand this framework, thus contributing theoretical guidance to the progression of the tourism industry. (2) Interconnectedness in Tourism Economy. The tourism economy exhibits substantial interconnectedness, and there may be additional factors within scenic area selection policies that potentially influence its development. Subsequent scholars might embark on a more comprehensive investigation of the influencing mechanisms within tourism economic development, furnishing support for its advancement. (3) Focus on 5ATAs Selection. The current inquiry solely centers on the impact and intrinsic mechanisms of 5ATAs selection on tourism economy, leaving many other selection policies unexplored. Ongoing attention to these policies in future research may offer valuable insights and references for the formulation and effectiveness of tourism industry policies. (4) Data and Conclusion Applicability. The present study harnesses data from China’s prefecture-level cities to scrutinize the ramifications of 5ATAs selection on the tourism economy. The inferences derived might not be universally applicable to selection policies abroad. Future researches are encouraged to integrate policies and pertinent data from diverse nations.

## Supporting information

S1 Data (XLS)
